# Enhancing Heat Transfer Behaviour of Ethylene Glycol by the Introduction of Silicon Carbide Nanoparticles: An Experimental and Molecular Dynamics Simulation Study

**DOI:** 10.3390/molecules28073011

**Published:** 2023-03-28

**Authors:** Xianjun Hou, Chen Chu, Hua Jiang, Mohamed Kamal Ahmed Ali, Karl D. Dearn

**Affiliations:** 1 Hubei Key Laboratory of Advanced Technology for Automotive Components, Wuhan University of Technology, Wuhan 430070, China; 2 Mechanical Design and Innovation, Department of Mechanical Engineering, School of Engineering, University of Birmingham, Edgbaston, Birmingham B15 2TT, UK; 3 Automotive and Tractors Engineering Department, Faculty of Engineering, Minia University, El-Minia 61519, Egypt

**Keywords:** thermophysical performance, interface effect, molecular dynamic, nanofluids

## Abstract

As the critical component of automotive engine coolant, ethylene glycol (E.G.) significantly matters in heat dissipation. In this study, the key aim is to investigate the heat transfer behaviour of E.G. as nano-additives base fluid. The heat transfer capability of E.G./SiC nanofluid (N.F.) was experimentally and theoretically evaluated via transient hot wire methods and equilibrium molecular dynamics (EMD) simulation, respectively. M.D. simulation exhibited a great ability to accurately forecast the thermal conductivity of N.F. compared with the experiment results. The results confirmed that the thermal stability of N.F. is relatively greater than that of E.G. base fluids. An improvement mechanism of thermal conductivity and thermal stability under an atomic scale via the analysis of mean square displacement (MSD) and radial distribution function (RDF) calculation was elaborately presented. Ultimately, the results indicated that the diffusion effect and the increasing transition rate of liquid atoms are responsible for thermal conductivity enhancement.

## 1. Introduction

Fluids with excellent heat transfer performance are urgently required due to the continuous development in industry and academia fields [1]. In the absence of nano-additives, the base liquid is thermally unstable at high temperatures and prone to oxidative decomposition [2,3]. In addition, in terms of heat transfer behaviour, the thermal conductivity of fluids is much lower due to the lack of control factors compared with solid materials [4]. In order to overcome this defect, N.F. arises accordingly [5,6]. As an emerging heat and mass transfer medium, N.F. is crucial in thermophysical properties. Multiple researchers have extensively investigated the heat transfer characteristics of N.F. They have great potential, receiving a great deal of research attention, such as electrochemistry [7,8], optics [9,10], pharmacology [11], solar energy [12,13], tribological lubrication [14,15,16]. One of the most significant indicators of thermophysical properties is thermal conductivity. Researchers have conducted various research on N.F.’s thermal conductivity, including experimental investigation and simulation analysis. However, the improvement mechanism of thermal conductivity from the interfacial effect of colloids to Brownian motion is of somewhat ambiguousness and insufficiency due to the complicacy of several effect factors such as temperatures, the type of base fluids, N.F. concentrations, size and shape of nanoparticles.

Temperature and nanoparticle concentrations are critical physical quantities affecting fluid’s thermal conductivity because they are closely related to Brownian motion and other important thermal factors [17,18,19,20]. From the particle motion level, with the increase in temperature, the Brownian motion further intensifies, causing collisions between atoms, and increasing the thermal conductivity. This conclusion is drawn by some researchers [21,22,23]. However, different researchers have put forward varying opinions. Jiang et al. [24] investigated the h-BN/PAO6 N.F. heat transfer performance under various temperatures and concentrations using a transient hot wire method. Eventually, the results suggested that the thermal conductivity of pure PAO 6 and N.F. with lower concentrations tends to decrease with increasing temperatures. In comparison, it is positively related to the rising temperatures for N.F. with higher concentrations due to the small size effect of h-BN nanoparticles. ZnO-TiO_2_/E.G. hybrid N.F.s were fabricated, and the heat transfer of the hybrid N.F.s was studied by Toghraieeret al. [25], who indicated that there was a positive correlation between the thermal conductivity of N.F.s and temperatures as well as volume fraction of nanoparticles. In addition, Li et al. [26] presented an investigation concerning the effect of particle concentrations and temperatures on the thermal conductivity of silica/E.G. N.F.

In addition to experimental investigation of thermal conductivity, regression fit analysis was utilized to predict thermal conductivity, including the most famous Maxwell and Einstein models. However, such simulation analysis based on experimental data can only speculate or estimate the underlying mechanism without firm evidence. Fundamentally, the key reason for the increase in thermal conductivity cannot be well analysed from the microscopic level. Conversely, molecular dynamic simulation can reasonably predict the thermal conductivity and analyse the microscopic atomic motion to achieve convincing mechanisms. Non-equilibrium molecular dynamics (NEMD) and EMD methods were the widely used two simulations for evaluating the heat transfer characteristics of N.F. NEMD simulation was adopted by Zhou et al. [27] to investigate and compare the heat transfer behaviour of Cu-Ar N.F. with and without agglomerated nanoparticles. The results showed that the interfacial solid–liquids interaction is responsible for the domination of the Ar atom on the decision of thermal conductivity. The RDF further confirmed that the aggregated particles are not conducive to the thermal conductivity improvement of N.F.s. In addition, Abu-Hamdeh et al. [28] studied the heat transfer peculiarities of Cu/water systems via the M.D. simulation approach. Ultimately, the consequences exhibited that the interaction between nanoparticles and surfactant facilitates the positive enhancement of thermal conductivity through a RDF analysis.

E.G., as the main component of automobile coolant, is critical to the cooling system of automobile engines because the heat dissipation performance of the engine was related to the E.G. fluids [29,30]. Therefore, it is necessary to improve the heat transfer characteristics of E.G. It is well known the heat transfer characteristics of nanofluids can be better improved than those traditional fluids without nanoparticles [31,32,33,34]. In addition, hydrothermal behaviours have been investigated by previous study [35,36], where they laid a solid foundation for heat transfer of N.F. SiC due to the great physicochemical performance was considered as the potential additive into the E.G. solution. In this paper, the thermal conductivity of E.G./SiC N.F.s is studied by M.D. simulation, and the potential mechanism for improving the thermal conductivity is given in detail. Simultaneously, the thermal conductivity is measured experimentally by the transient hot wire method and compared with the M.D. simulation results. Finally, the simulation results agreed well with the experimental results, and the results showed that SiC nanoparticle contributes to the increase in the thermal conductivity of E.G., and the most convincing reason for its improvement is analysed from the atomic level.

This paper aims to reveal several thermal conductivity improvement mechanisms for N.F.s with diverse concentrations at varying temperatures. Thus, the EMD means was employed to model and simulate the glycol/SiC N.F. system and the heat transfer performance was discussed. It was profoundly revealed the diffusion behaviour of SiC nanoparticles in E.G. from the micro scale. Eventually, the active adsorption layer between SiC and E.G. base liquids promoted the improvement of thermal conductivity.

## 2. Results and Discussion

### 2.1. Characterization of SiC Nanoparticle

**Figure 1:**
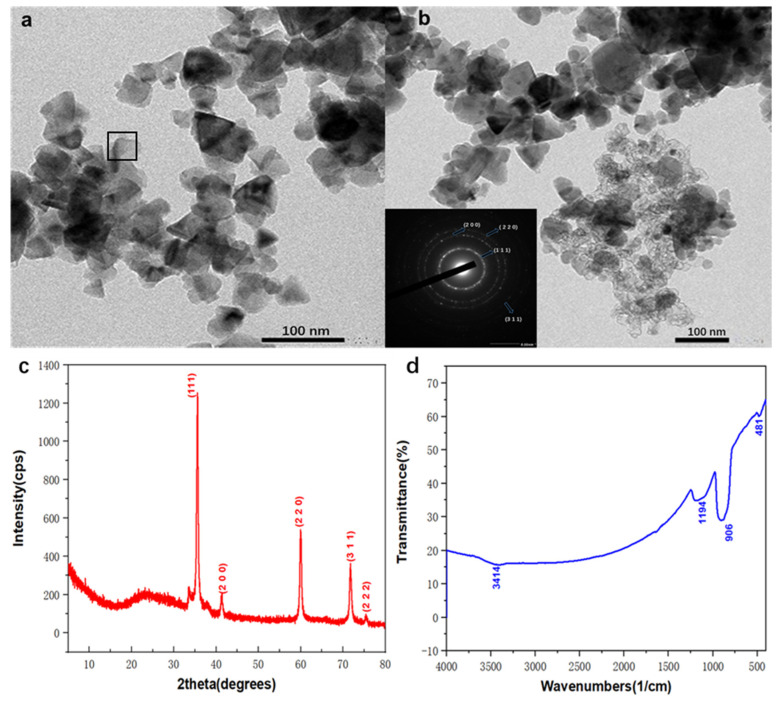
A series of original characterizations of SiC nanoparticles, (**a**,**b**) TEM and SAED, (**c**) XRD, (**d**) FTIR.

For the exploration of the morphology and size distribution of SiC nanoparticles used, TEM as well as SAED characterisation under different locations, were conducted in Figure 1a,b, in which most of the particles distributed from 40–60 nm, and the average size remains approximately 50 nm, which agreed well with the label used in Table 1 and confirmed the nano size of SiC particles. A few nanoparticles were greatly agglomerated into large clusters. Furthermore, electron diffraction (SAED) pattern at a chosen area was recorded from a marked location in Figure 1a, indicating the polycrystalline characteristics possessing superior crystallinities. XRD characterisation of SiC was detected in Figure 1c. Five prominent characteristics peaks were noted at the two theta of approximately 35.4, 40.05°, 60.12°, 71.9° and 75.8°, which can be ascribed to the (111), (200), (220), (311), and (222) crystal face of SiC phase and is highly in line with the results of JCPDS, No. 29–1129 [37]. The XRD results confirmed the high purity of the SiC nanoparticles used in this study. In addition, the FTIR characterisation of SiC nanoparticles was exhibited in Figure 1d, where four characteristic peaks can be observed. Si(OH)n characteristic vibration is responsible for the appearance of transmittance peaks located at about 481cm^−1^ [38]. The transmittance stretching band noted at the wavenumbers of approximately 906 cm^−1^ may be assigned to the C-Si vibration mode of SiC. In addition, the stretching vibration peak at approximately 3414 cm^−1^ may be attributed to the O-H formation from the water molecule in the air.

### 2.2. Thermal Stability of N.F.s

**Figure 2:**
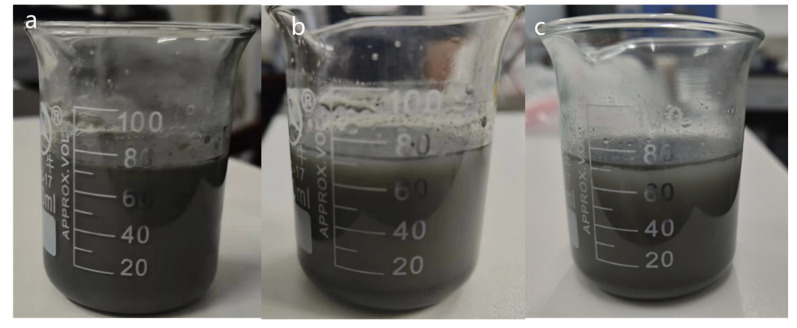
The sedimentations images of 0.5% SiC N.F.s at different time, (**a**) 1 day (**b**) 3 days (**c**) after 5 days.

**Figure 3:**
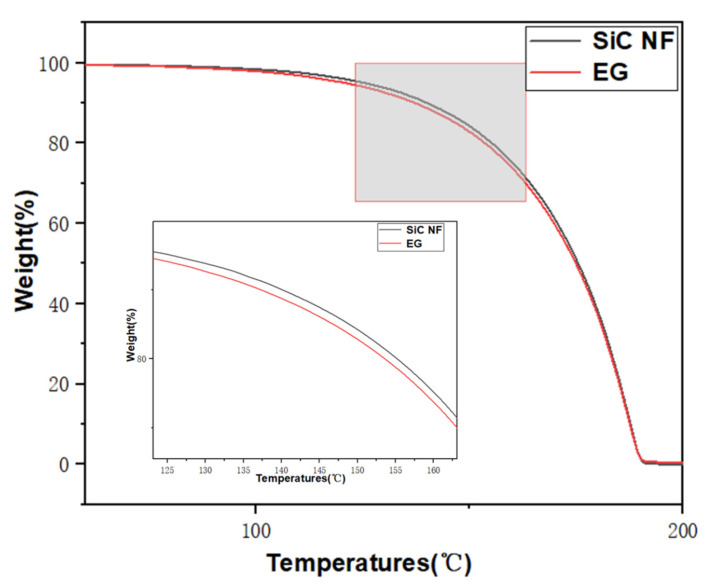
TGA analysis of E.G. and SiC N.F.

Figure 2 exhibited the natural sedimentations image of SiC N.F. On the first day, the whole N.F. system was stable and evenly dispersed, and no agglomerations and sedimentations were observed visually. Over time, sedimentations were observed on the third and fifth day, which may be related to the van der Waals attraction between nanoparticles. In the subsequent research, it can be functionalized to enhance the electrostatic repulsion between nanoparticles and prevent the agglomerations of particles. In addition to thermal conductivity, thermal stability is also an essential part of the thermophysical properties of N.F. Improving the unstable state of fluids at high temperatures due to oxidative decomposition contributes to an augment in their thermal conductivity. Figure 3 shows TGA tests of base liquid and SiC N.F. The results indicated that the TGA curve of SiC N.F. was higher than that of E.G., which means SiC N.F. lost less mass at the same temperature, confirming the relatively better thermal stability of SiC N.F. compared with the E.G. alone.

### 2.3. Thermal Conductivity of E.G./SiC N.F.

**Figure 4:**
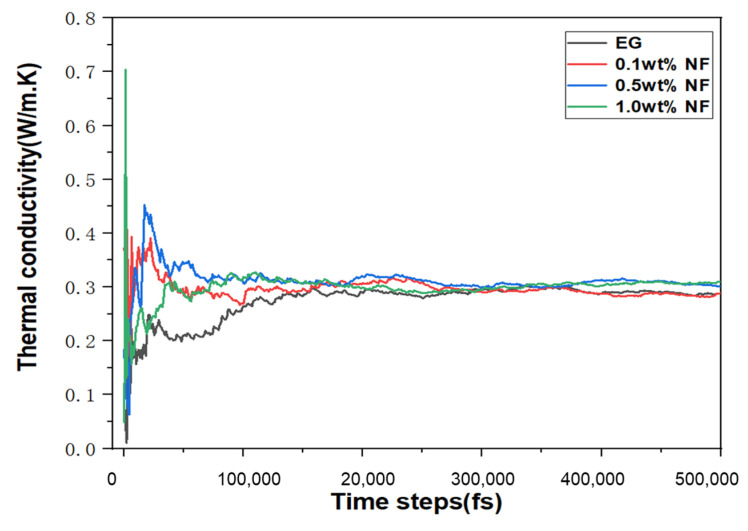
The thermal conductivity -approaching equilibrium over time steps of 500,000 fs.

**Figure 5:**
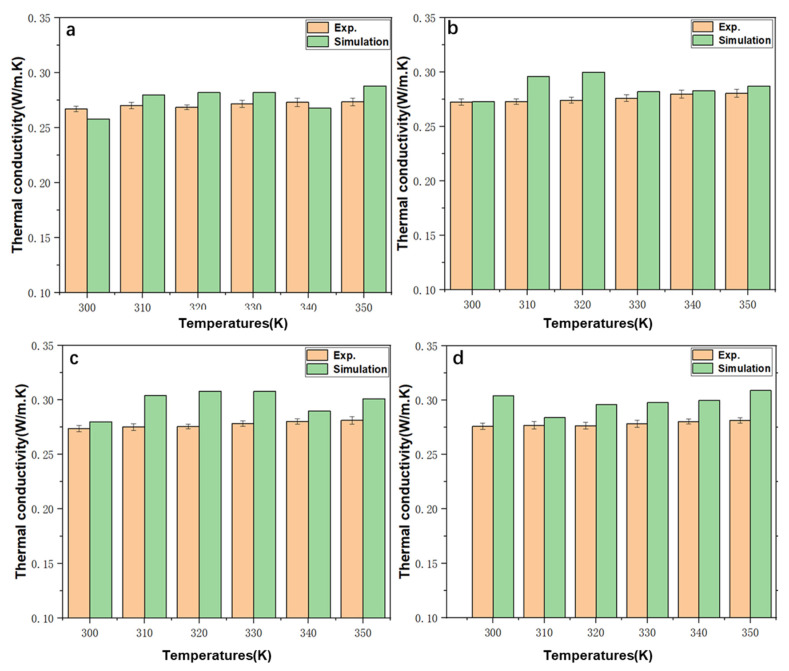
The experiment and simulation values of thermal conductivity of (**a**) E.G., (**b**) 0.1% N.F., (**c**) 0.5% N.F., (**d**) 1% N.F, under varying temperatures.

The thermal conductivity of pure E.G. and 0.1–1% N.F. at different temperatures were derived by the EMD simulation approach and compared with the experiment results using transient hot wire methods. Figure 4 presented the correlations between dynamic thermal conductivity change and time steps during the M.D. simulation process. There were apparent fluctuations for all specimens before approaching a balanced state. Notably, the thermal conductivity of E.G. converges at a time step of about 100,000 fs, while the thermal conductivity of N.F. reaches thermal equilibrium at about 500,000 fs. The simulation and experimental values of thermal conductivity are exhibited in Figure 5, where the effects of nanoparticle concentrations and the temperatures on the thermal conductivity of base liquids are elaborately investigated. Figure 5a–d shows the experimental and simulation values of the thermal conductivity of E.G., 0.1% N.F.s, 0.5% N.F.s and 1% N.F.s, respectively. It can be distinctly noted that the results show that the thermal conductivity of most samples increases with temperature, except the several datum that slightly decreased with temperatures. In addition, thermal conductivity of N.F.s with SiC nanoparticles is higher than that of E.G. at the same temperature. The simulation results show that the overall thermal conductivity also increases with the increasing temperature, except that the N.F. at a specific temperature, where the thermal conductivities, in turn, drops with the rising temperatures. Additionally, similar to the experimental results, the thermal conductivity of N.F.s is significantly higher than that of pure E.G. liquids. For instance, the simulation value of thermal conductivity for pure E.G. under 350 K is 0.288, while 0.309 for 1% N.F. The increasing rates of experimental and simulated values of N.F. thermal conductivity over E.G. are 5.4% and 7.3%, respectively. More importantly, whether it is E.G. or N.F. with varying molar fractions, the thermal conductivity simulated by M.D. approaches the experimental value with lower error, further illustrating the positive effectiveness and feasibility of the M.D. model adopted in this study. Furthermore, the 1.0% N.F.s exhibited optimal thermal conductivity compared with other samples, jointly confirmed by experimental and simulation results.

**Figure 6:**
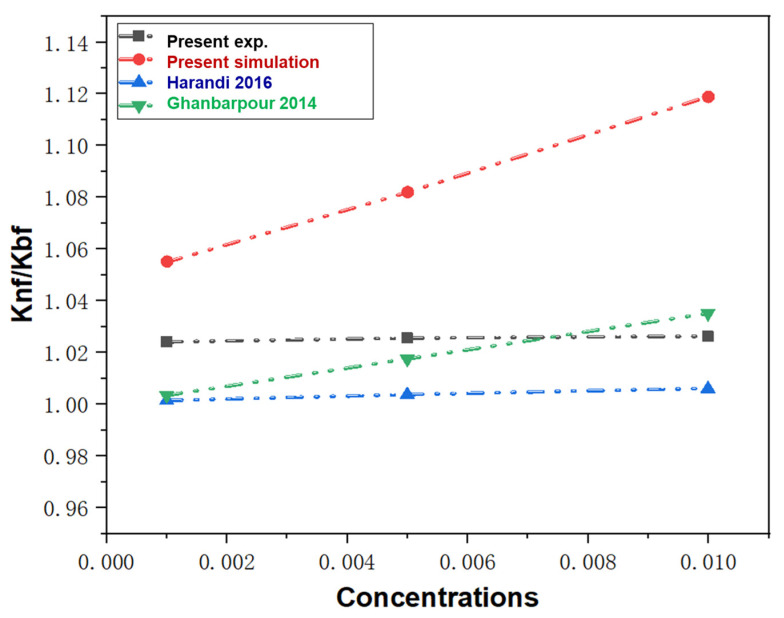
The exp. and simulation value of relative thermal conductivity of N.F. with different concentrations compared with previous investigations [39,40] at the temperatures of 340 K.

Figure 6 exhibited the current experiment and simulation values of thermal conductivity of various concentrations N.F. compared with the previous study results. All these curves indicated that the trend of relative thermal conductivity increases with the augment of N.F. concentrations. The experimental value is very close to the previous research results [39,40], where regression analysis is used to predict the thermal conductivity of N.F. All thermal conductivity ratios range from around 1 to 1.12. In general, the experimental and simulation data of thermal conductivity in this paper are similar to the results of the previous two studies, that is, the thermal conductivity increases with the increase in concentration. Although the M.D. simulation results are slightly higher than the experimental results, the change trend is similar. The reason that the experiment values differ with the simulation results may be the controlling factors such as the potential function selection, the parameters settings, and the system error. In addition, the Knf/Kbf values from our experiment, M.D. simulation, and two previous investigations are 1.02416, 1.055, 1.0015, 1.0035, respectively. It also proves the credibility and effectiveness of the experiment and simulation results.

### 2.4. MSD Analysis of the N.F. Systems

The MSD is used to measure the bias between the location of particles’ motion over time. This phenomenon is a standard method to analyse the dynamic characteristics of the N.F. system. The formula for calculating MSD is as follows:

$$MSD\left(t\right)=\frac{1}{N}\langle \sum_{i=1}^{N}{\left|r_{i}\left(t\right)-r_{i}\left(0\right)\right|}^{2}\rangle$$

where N is the number of particles, *t* is the time, $r_{i}\left(t\right)-r_{i}\left(0\right)$ is the vector distance that a given particle passes through in a period of time.

**Figure 7:**
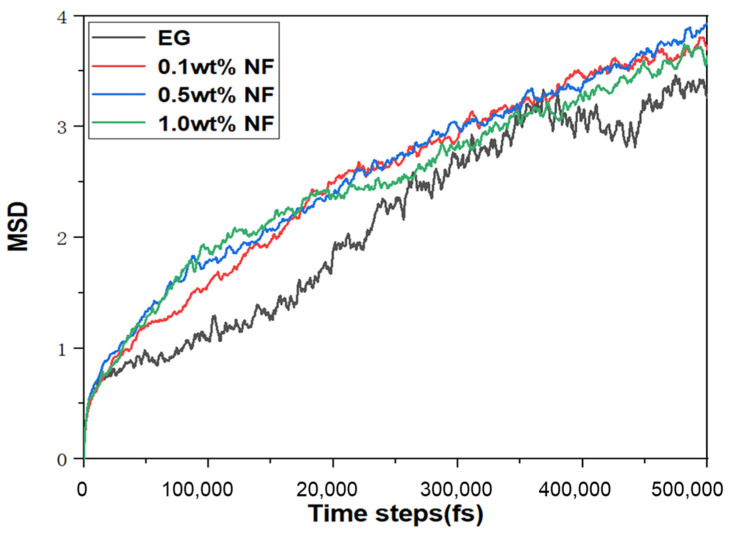
The MSD curve of E.G., 0.1%, 0.5%, and 1.0% N.F.

**Figure 8:**
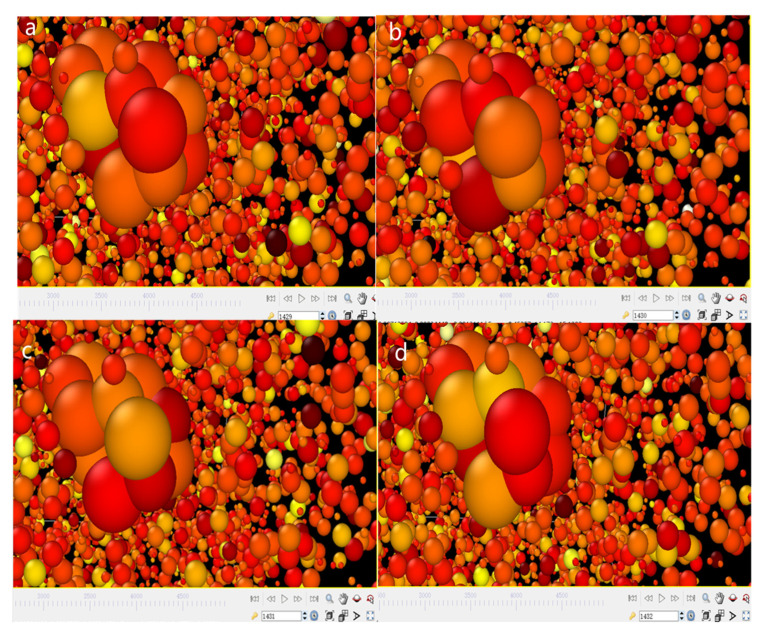
The micro atom motion status under different time steps, (**a**) 1429 ps, (**b**) 1430 ps, (**c**) 1431 ps, (**d**) 1432 ps.

The diffusion coefficient can be calculated to account for the aggregation and adhesion of the nanosystem. The MSD curves of the total atoms in the whole E.G./SiC N.F.s system with various molar fractions under diverse temperatures are depicted in Figure 7. The calculation duration required for MSD is set as 500,000 fs. We can observe that the curve slope of the primary liquid E.G. is the lowest in the total MSD value, while the slope of 1% N.F. is the highest. Consequently, the motion displacement of pure E.G. appears lower than that of 0.1–1% N.F.s, revealing that the diffusive rate of SiC N.F. is higher than E.G. base fluid, which is similar with the results of the previous study [41]. In the N.F.s system, liquid atoms move much faster than SiC nanoparticle atoms. The rapid increase in movement velocity is because of the addition of nanoparticles. Therefore, the significantly improved liquid atom motion contributes to effectively transferring heat and enhancing thermal conductivity in the N.F.s system, which is also consistent with the findings of previous investigations [42]. Pronounced anisotropy can be observed in the MSD curves, which indicates that the potential energy interaction between nanoparticles and E.G. molecules is highly correlated with the diffusion performance of the whole system. It is also indirectly reflected in the improvement of the transition ability of nanoparticle atoms after the addition of SiC nanoparticles, which stimulates the migration rate of liquid atoms. Moreover, the results have confirmed the contribution of SiC nanoparticles in improving liquids atoms motion velocity and enhancing the probability of nanoparticles collision, which leads to an augmentation of the thermal conductivity of N.F.s. In addition, Figure 8 showed the micro atom motion state under consecutively varying time steps. It can be noted that the dark colour of the atoms denotes the higher temperatures. In the simulation, the temperature stands for the velocity, but for a single atom, the velocity is fluctuating. From 1429 to 1432 ps, we can see in Figure 8 that the colour of liquids atom are darker than the solid, confirming the quicker velocity of liquids atom. 

### 2.5. RDF Analysis and Atom Motion Trajectory for N.F.

**Figure 9:**
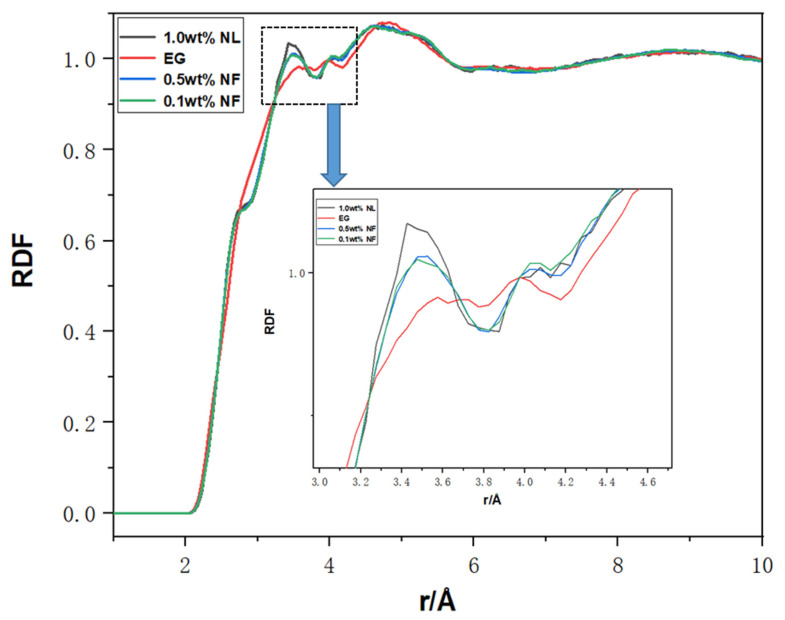
The total RDF of pure E.G. and various concentrations N.F.s.

The RDF reflects the fluid microstructure and reveals the aggregation behaviour of different atoms in the fluid. Figure 9 shows the RDF of pure E.G. and N.F.s with different molar fractions. Additionally, the influence of the two parameters (temperature and molar fraction) on the thermal conductivity of N.F. are accurately reflected in the values of RDF. Higher concentration N.F. tends to possess higher RDF value, which shows that the nanolayer presents a more compact solid state due to the effect of nanoparticles. In addition, as the figure presented, three peaks of the RDF function are visually found. With the augment of r, the RDF value subtly fluctuates prior to accessing a stable value of 1, which asserts that the structure of the outermost molecular nanolayer is arranged in order, and the interaction between SiC molecules and, E.G. molecules appears the strongest. Due to the significant surface energy and specific surface area on the surface of SiC nanoparticles, the nano layer’s interfacial effect and the particles’ small size effect are strengthened during the heat conduction process of N.F.s. In addition, the increase in N.F.s molar fraction results in a decrease in the contact thermal resistance due to activation surface free energy. In diffusion behaviour, the influence of the adsorption layer is crucial because the spatial structure of the adsorption layer of E.G. base solution and N.F.s differs, which confirms the difference in their thermal conductivity. Therefore, the heat flow density will also increase accordingly in the heat transfer process, improving thermal conductivity. In addition, due to the diffusive behaviour in the interfacial effect, the absorption layer is also a potential cause for deciding the thermal conductivity [43]. RDF results showed that the probability of finding another atom around one atom in SiC N.F.s is significantly higher than that of E.G. basic fluids. It can be concluded that the addition of SiC nanomaterials changes the microstructure of basic fluid E.G. This makes the microstructure of the pure liquid phase of the base liquid change towards to that of the solid–liquid phase, thus enhancing the thermal conductivity.

**Figure 10:**
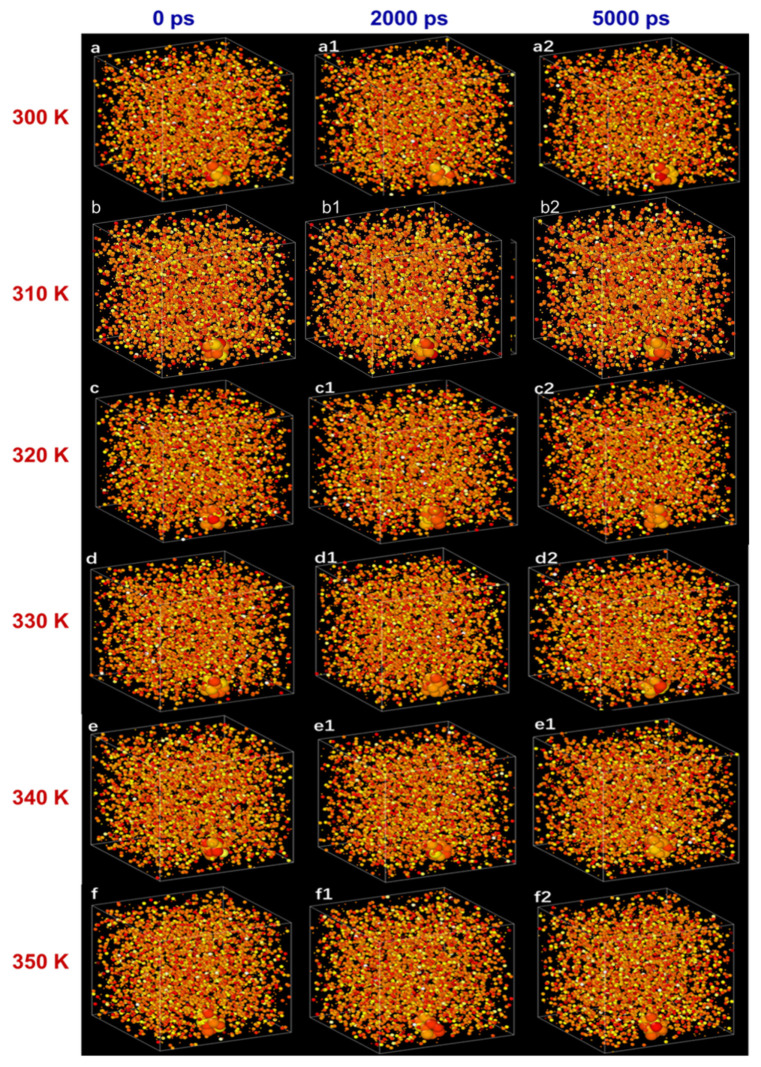
The atom thermal motion status of 1% SiC N.F. under varying time steps at different temperatures. (**a**,**a1**,**a2**) at the temperature of 300 K, time steps of 0 ps, 2000 ps, and 5000 ps. (**b**,**b1**,**b2**) at the temperature of 310 K, time steps of 0 ps, 2000 ps, and 5000 ps. (**c**,**c1**,**c2**) at the temperature of 320 K, time steps of 0 ps, 2000 ps, and 5000 ps. (**d**,**d1**,**d2**) at the temperature of 330 K, time steps of 0 ps, 2000 ps, and 5000 ps. (**e**,**e1**,**e2**) at the temperature of 340 K, time steps of 0 ps, 2000 ps, and 5000 ps. (**f**,**f1**,**f2**) at the temperature of 350 K, time steps of 0 ps, 2000 ps, and 5000 ps.

**Figure 11:**
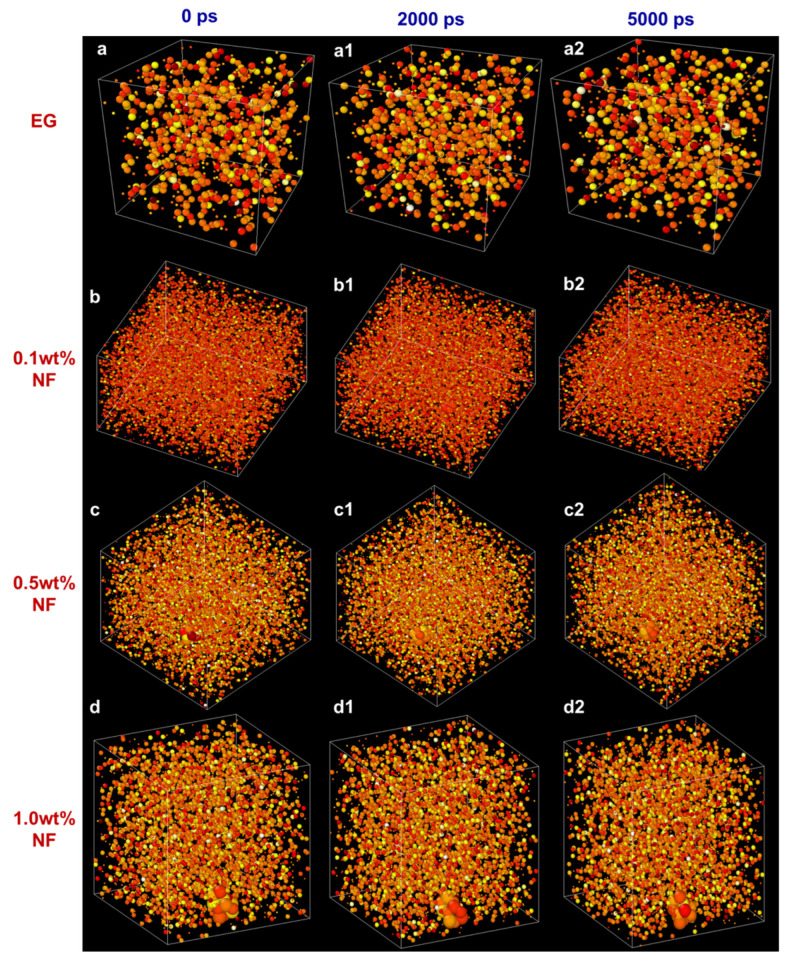
The atom thermal motion status under varying time steps at different N.F.s concentrations under 350 K. (**a**,**a1**,**a2**) E.G., time steps of 0 ps, 2000 ps, and 5000 ps. (**b**,**b1**,**b2**) 0.1% N.F., time steps of 0 ps, 2000 ps, and 5000 ps. (**c**,**c1**,**c2**) 0.5% N.F., time steps of 0 ps, 2000 ps, and 5000 ps. (**d**,**d1**,**d2**) 1.0% N.F., time steps of 0 ps, 2000 ps, and 5000 ps.

Figure 10 depicts a motion path of atoms surrounded by N.F. at different temperatures (300–350 K) for different time steps (0, 2000, 5000 ps). As the time step changed, the atomic motion is disorderly, and no rule seems to be found. It can be noticed that with the increase in temperature, the number of atoms with darker colour in the diagram increases, showing both liquid atoms and SiC nanoparticle atoms move more intensely due to the increase in Brownian motion, but the atoms of nanoparticles move more slowly than those of liquids atoms in E.G. Moreover, Figure 11 shows the atom motion trajectory of E.G. and N.F. In the E.G. base liquid system, the liquid atoms move continuously with the increasing time step. However, because there is no control factor (i.e., nanoparticles), the adsorption layer formed during the moving process is not firm and cannot form an excellent thermal conductivity channel, so its thermal conductivity is relatively low. Conversely, in the N.F. system, with nanoparticles additions into the E.G. base fluid, it can be found that the nanoparticles strongly adsorb around the liquid atoms, forming a denser adsorption layer structure at the solid–liquid interface and enhancing the diffusion effect of the base liquid. These factors may be responsible for the enhancement of thermal conductivity.

## 3. Computational M.D. Simulation and Experimental Section

### 3.1. The Description of the M.D. Simulation

Molecular dynamics, a multidiscipline technology combining mathematics, physics and chemistry, is a simulation method using Newtonian mechanics to simulate and calculate the motion state of microscopic molecule for obtaining a series of macroscopic physical quantities, such as thermal conductivity, friction coefficient, dynamic viscosity, velocity, temperature, shear stress and other parameters. With the development of simulation, M.D. simulation has been gradually adopted by multiple researchers to remedy the shortcomings of experimental methods. The movement law of atoms was accurately detected by M.D. analysis, and it elucidated the reasons for the improvement of dispersion behaviour, rheological behaviour, thermal performance, and friction performance from the atomic scale. This paper uses the open-source software LAMMPS 64-bit 29 October 2020 (Sandia National Laboratories, Albuquerque, NW, USA). A large-scale Atomic/Molecular Massively Parallel Simulator was considered for the simulations to investigate the heat transfer characteristics of pure E.G. and E.G./SiC N.F.s with different concentrations.

### 3.2. Inter-Atomic Potentials and Simulation Strategy

**Figure 12:**
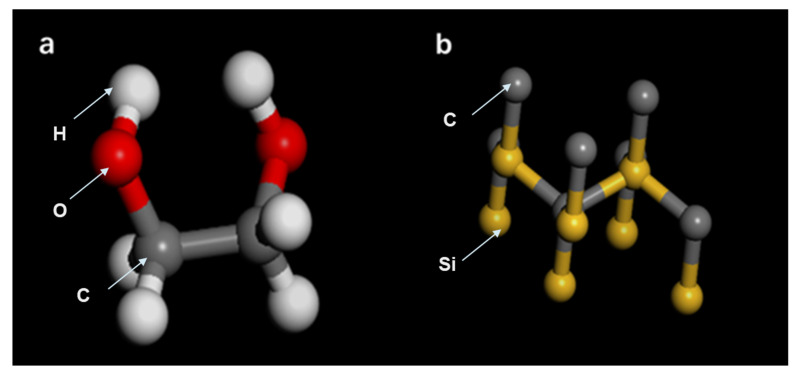
The Molecular unit cell of (**a**) E.G. and (**b**) SiC.

**Figure 13:**
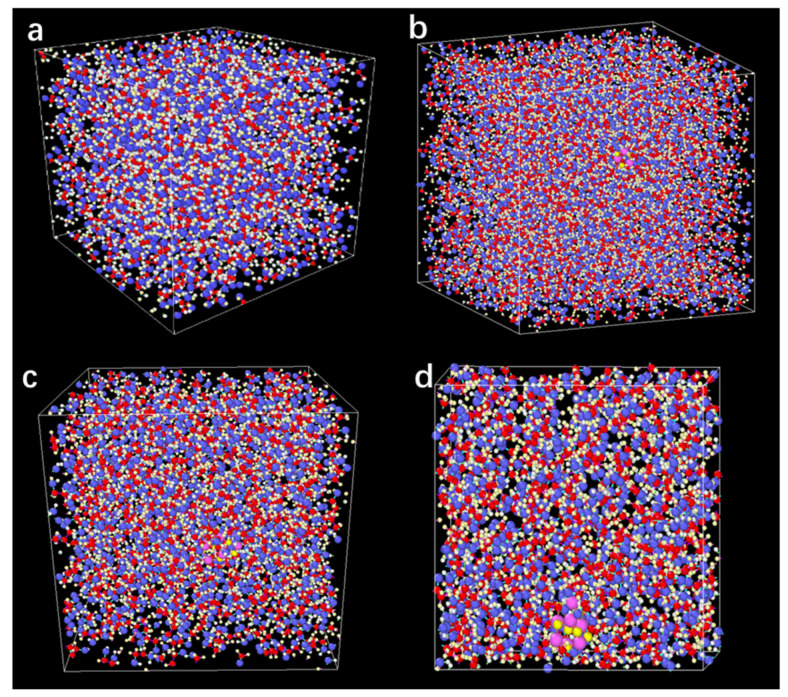
The MD model of E.G. and N.F. with concentration of (**a**) E.G., (**b**) 0.1%, (**c**) 0.5%, and (**d**) 1%.

Prior to the model construction, the SiC and E.G. structure cells can be seen, as shown in Figure 12. In the initial simulation stage, the required simulation systems of pure E.G. and E.G./SiC N.F. are established, respectively. First, the molecular unit cells required for the model building of SiC and E.G. are shown in Figure 12, in which individual atoms’ composition and arrangement order can be distinctly seen. In addition, as exhibited in Figure 13, the simulation models of E.G./SiC N.F.s with a molar fraction of 0%, 0.1%, 0.5%, and 1% are built. The larger particles are SiC N.P.s, and the dense linear molecules are E.G. molecules. A simulation box surrounds all models, and the steric sizes of the simulation box are 21.1 Å × 21.1 Å × 21.1 Å in the x-, y-, and z- orientations, respectively. The total model atom number required for the M.D. simulation is listed in Table 2.

To achieve the reliability, creditability, and precision of the simulation results, several force fields between atoms are carefully chosen. Lennard-Jones (LJ) Long range Coulomb force potential for C-C, O-H, Si-Si interactions with a cut-off distance of 10 Å was used. This force field is suitably extended on the basic of LJ potential, which increases the Coulomb pair interaction between atoms, making the output results more accurate. The formula of this force field can be expressed as follows:

(1) $$E=4\epsilon \left[{\left(\frac{\sigma }{r}\right)}^{12}-{\left(\frac{\sigma }{r}\right)}^{6}\right]\;r<r_{c}$$

(2) $$E=\frac{Cq_{i}q_{j}}{\epsilon r}\;r<r_{c}$$

where C is an energy-conversion constant, $q_{i}$ and $q_{j}$ are the charges on the 2 atoms, and ϵ is the dielectric constant set by the dielectric command, which reflects the strength of the interaction between two atoms. σ is the distance between atoms when the action potential is equal to 0. The specific atom parameters value can be found in Table 3. In addition, due to the interaction of chemical bonds (C-C, C-H, O-H, C-Si, C-O), bond angles (C-Si-C, Si-C-Si, H-C-H, O-C-H, C-C-H, C-C-O, etc.), the harmonic potential function is used, which can be expressed as follows:

$$E1=K1{\left(r-r_{0}\right)}^{2}$$

$$E2=K2{\left(\theta -\theta_{0}\right)}^{2}$$

where *r* is the equilibrium bond distance, E1 and E2 are the stretching and bending energy, θ is the equilibrium value of the angle, and K1 and K2 are prefactors of bond and angle, respectively. The detailed bond and angle parameters values of harmonic potential used are summarized in Table 4 and Table 5. 

First, the entire simulation system needs to be balanced for several ns to obtain a constant temperature of 300–350 K in the NVT ensemble using the Nose Hoover thermostat. Then, EMD operation is conducted in the NVE ensemble to calculate the thermal conductivity and heat flux. In this EMD method, the thermal conductivity is calculated by heat flux. The method for calculating the heat flux is based on the contribution of atoms in the designated group. It can be used to measure the heat flow through a group of atoms (for example, the area between two constant temperature reservoirs at different temperatures), and the thermal conductivity can be calculated by Green Kubo formula as follows:

$$\begin{array}{cc} & J=\frac{1}{V}[\underset{i}{\sum }\;e_{i}v_{i}-\underset{i}{\sum }\;S_{i}v_{i}]\\ & =\frac{1}{V}[\underset{i}{\sum }\;e_{i}v_{i}+\underset{i<j}{\sum }\left(F_{ij}\cdot v_{j}\right)r_{ij}]\\ & =\frac{1}{V}[\underset{i}{\sum }\;e_{i}v_{i}+\frac{1}{2}\underset{i<j}{\sum }\left(F_{ij}\cdot (v_{i}+v_{j})\right)r_{ij}] \end{array}$$

$$\kappa =\frac{V}{k_{B}T^{2}}\int_{0}^{\infty }\left\langle J_{x}(0)J_{x}(t)\right\rangle dt=\frac{V}{3k_{B}T^{2}}\int_{0}^{\infty }\langle J(0)\cdot J(t)\rangle dt$$

where *V* denotes the volume, *k_B_* denotes the Boltzmann constant, *T* is the temperature, <*J_x_*(0)*J_x_*(*t*)> is the heat flow autocorrelation function.

### 3.3. Verification of the M.D. Simulation Model

For the detection of M.D. model effectiveness, the equilibrium relaxation of E.G./SiC N.F.s with different molar fractions at different temperatures under initial conditions was calculated between the time steps of 0–250 ps. In this investigation, the thermal conductivity of E.G. was simulated by the EMD method. The CVFF force field is used in the simulation research system. The molecular optimisation function determines the bond length and angle of the E.G. molecule. Finally, SiC cells are introduced to obtain E.G./SiC models with molar fractions of 0%, 0.1%, 0.5%, and 1%. The conjugate gradient method eliminates unreasonable interactions in the system and minimises the energy. Then, the Nosé Hoover hot bath method is used to relax for 1 ns under the constant pressure and temperature (NPT) ensemble. The temperature gradient is accordingly set (300 K, 310 K, 320 K, 330 K, 340 K, and 350 K). The pressure is set as standard atmospheric pressure. Then, after withdrawing the NPT ensemble, the simulation was relaxed for 1 ns under the constant volume and temperature (NVT) ensemble. When the whole system reached equilibrium, the NVT ensemble was removed. The system finally runs 200 ps under the micro-canonical ensemble (NVE) to obtain the kinetic energy, potential energy, and potential force of atoms. The heat flow density through the energy contribution of atoms in the group is calculated and sampled every 1000 steps. Finally, we connect the heat flow density J with the autocorrelation set average of the thermal conductivity k through the Green-Kubo formula, and the thermal conductivity is accordingly calculated. The time step is set as 1 fs. Periodic boundary condition (PBC) is chosen in the simulation system, the Leap Frog algorithm is used for the equation of motion integration, and the long-distance electrostatic interaction calculation needs to be matched with PPPM (10^−4^ Å) in Lammps. Ovito software (University of Freiburg, Germany) was used to post process the simulation results.

### 3.4. Experimental Section

#### 3.4.1. Materials

SiC nanoparticles were purchased from a commercial company (Shanghai Chaowei Nanotechnology Co., Ltd., Shanghai, China), and E.G. is obtained from Shanghai Aladdin Bio-Chem Technology Co., Ltd., Shanghai, China. The detailed physical parameters of SiC nanoparticles were displayed in Table 1.

**Figure 14:**
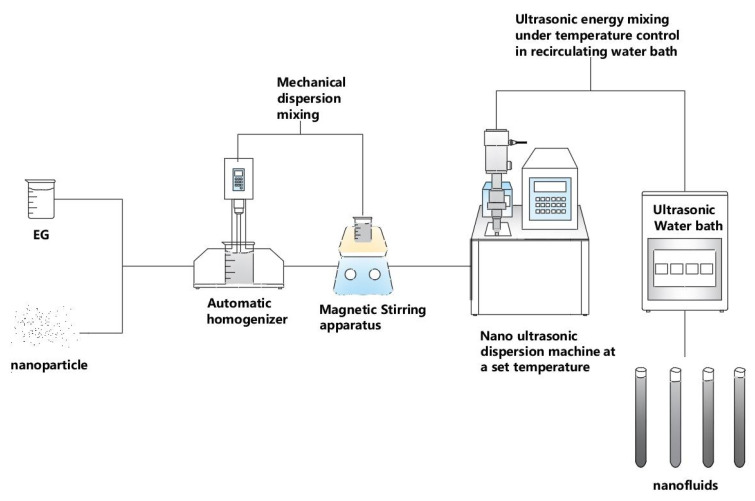
The specific fabrication procedure of N.F. via a two-step means.

In the preparation of N.F.s, a conventional two-step method was applied. First, SiC nanoparticles were added into E.G. base fluid, and the mechanical dispersion, including high-pressure homogeniser and magnetic stirrer was used. Under the premise of mechanical stability, ultrasonic dispersion is carried out to further improve its dispersion stability, including ultrasonic probe and ultrasonic bath dispersion. In this way, stable N.F.s are well prepared. The detailed preparation process is shown in Figure 14.

#### 3.4.2. Equipment Employed

In nanoparticle characterisations, we use TEM (JEM-1400Plus, JEOL, Tokyo, Japan) to detect the particles’ average diameter, overall distribution, and aggregation conditions in E.G. fluids. XRD (Empyrean, PANalytical, Almelo, The Netherlands) and FTIR (Nicolet 6700, Thermo Fisher Scientific, Waltham, MA, USA) were used for the crystal construction, chemical bonds, and micro-valence change. In the manufacturing process of N.F.s, a high-pressure homogeniser and magnetic stirrer are used to mix nanoparticles and base liquid physically. The ultrasonic energy generated by the ultrasonic disperser due to the cavitation role makes the N.F. uniform and stable. Finally, the prepared N.F. is put into the ultrasonic bath for re-stablisation. Thermal stability of N.F. and E.G. was assessed by TGA analysis conducted by Thermogravimetry meter (NETZSCH-STA449F3, Selb, Germany). Thermal conductivity meter (XIATECH, TC3000E, Xi’an, China) is experimentally used to measure thermal conductivities of N.F. and base fluid via a transient hot wire method. Each experiment was performed by 3 times and the average with standard deviations were taken.

## 4. Conclusions

This study explores the heat transfer capability of E.G. and reveals the enhanced mechanism of E.G. thermal conductivity by adding SiC nanoparticles via an EMD simulation under varying temperatures and concentrations. Based on the results and discussion, the primary conclusion can be presented as follows:

Thermogravimetric analysis confirmed that the SiC/E.G. N.F.s appear thermally stable compared with the E.G. alone, which is mainly attributed to the small size effect caused by SiC nanoparticles addition.

The E.G./SiC N.F. thermal conductivity was predicted and simulated using M.D. simulation. Most of simulation values of the thermal conductivity increased with the increasing temperature except the several contrary results. In addition, compared with the E.G. solution alone, thermal conductivity of SiC N.F. was improved, by up to approximately 5.4% and 7.3%, for experiment value and simulation value, respectively, which showed a credibility for simulation results.

The MSD curve proves that the addition of nanoparticles greatly accelerates the movement velocity of liquid atoms in N.F.s, widens the diffusion layer, and improves the diffusion efficiency. The interface effect and the soaring motion of liquid atoms are the fundamental reason, rather than the Brownian motion improvement, for improving thermal conductivity.

RDF analysis confirmed that the microscopic structure of E.G. base fluid changed from the liquid phase into a solid–liquid state due to the introduction of SiC nanomaterials, which may also be the reason responsible for the enhancement of thermal conductivity.

## Figures and Tables

**Table 1:** The physical parameters of SiC used.

Nanoparticles Used	Density (g/cm^3^)	Average Size (nm)	Specific Surface Area (m^2^/g)	Crystal Form
SiC	0.11	40–60	39.8	Cubic

**Table 2:** The number of molecule and atoms used.

SiC Molar Fraction	E.G. Molecule	Si Atom	C Atom	Total Atoms
0%	1000	0	0	10,000
0.1%	4470	7	7	44,714
0.5%	899	7	7	9004
1%	447	7	7	4884

**Table 3:** Detailed atom parameters required for LJ potential used.

Atom	σ (Å)	Ε (kJ/mol)	Molar Mass	Charge q (|e|)	Notes
C1 (SiC)	3.4745	0.159	12.0100	0.000000	C atom from SiC
Si	4.0534	0.2000	28.0860	0.000000	\
C2 (E.G.)	3.8754	0.1577	12.0100	−0.170000	C atom from E.G.
H1 (C)	2.4499	0.3038	1.0080	0.100000	H atom from -CH_2_
H2 (O)	2.4499	0.1569	1.0080	0.350000	H atom from -OH
O	2.8597	0.5803	16.0000	−0.380000	\

**Table 4:** Detailed bond parameters required for harmonic potential used.

Chemical Bond	*K*1	*r* (Distance)
C2-C2	322.7158	1.5260
C2-O	384.0000	1.4200
C2-H1	340.6175	1.1050
O-H2	540.6336	0.9600
C1-Si	238.0000	1.8090

**Table 5:** Detailed bond angle parameters required for harmonic potential used.

Angle	*K*2	θ (Degrees)
C2-C2-O	70.0000	109.5000
C2-C2-H1	44.4000	110.0000
O-C2-H1	57.0000	109.5000
H1-C2-H1	39.5000	106.4000
C2-O-H2 Si-C1-Si C1-Si-C1	58.5000 42.2000 44.4000	106.0000 122.5000 113.5000

## Data Availability

Not applicable.
